# Ti-Based Metallic Biomaterials for Antitumor Applications

**DOI:** 10.3390/ma18102262

**Published:** 2025-05-13

**Authors:** Xiang Yan, Hui Liu, Zhe Zhang, Xiang Deng, Manfeng Lin, Zongyuan Cai, Dongying Tang, Hang Wang, Wen Liu, Dapeng Zhao

**Affiliations:** 1 School of Information and Intelligent Engineering, Zhejiang Wanli University, Ningbo 315100, China; tcxy1986@163.com (X.Y.); wanghang@zwu.edu.cn (H.W.); 2 College of Biology, Hunan University, Changsha 410082, China; huiliu2024@outlook.com (H.L.); z1895@hnu.edu.cn (Z.Z.); dengxiang@hnu.edu.cn (X.D.); linmanfeng@hnu.edu.cn (M.L.); s2322w1872@hnu.edu.cn (Z.C.); dytang01@126.com (D.T.)

**Keywords:** Ti-based metallic biomaterials, antitumor, photothermal therapy, photodynamic therapy, local drug delivery

## Abstract

Titanium (Ti)-based metallic biomaterials (MBs) are traditionally employed as mechanical supports and constraints in clinical practice, owing to their superb comprehensive mechanical properties, great corrosion resistance, and good biocompatibility. Recently, Ti-based MBs have emerged as promising candidates for antitumor applications. These developments focus on the functionalization of Ti-based MBs to inhibit tumor propagation and recurrence. This work systematically examines the antitumor approaches of Ti-based MBs and categorizes them into physical and chemical approaches. Physical strategies, such as the photothermal and photocatalytic techniques, are usually related to material-specific properties. Chemical approaches often employ controlled local drug delivery (LDD) systems. Ti-based LDD systems enable the targeted release of chemotherapeutics, metal ions, or immunomodulatory agents at tumor sites. This review highlights the efficacy of these surface-functionalized Ti-based MBs against diverse tumors. Additionally, the challenges and prospects of antitumor Ti-based MBs are also discussed.

## 1. Introduction

Cancer remains a major public health problem worldwide [1]. Current clinical interventions, such as surgery, chemotherapy, and radiotherapy, are still constrained by systemic toxicity, short half-lives of chemotherapeutics, non-specific biodistribution, and acquired therapeutic resistance [2]. Consequently, increasing attention has been paid to innovative therapeutic strategies, which combine tumor-specific targeting with minimizing collateral damage to healthy tissues [3]. Over the past two decades, various biomaterials have been developed to overcome these limitations and exhibit their potential in oncotherapy through therapeutic drug delivery [4], immunomodulation [5], oxidative stress suppression [6], and so on. For example, nanoparticle biomaterials deliver drugs to target cancer cells via active and passive mechanisms [7,8,9]. However, the delivery system that nanomaterials travel throughout the body in the bloodstream increases the required dose, which results in various side effects and damages other tissues [10].

Owing to their good comprehensive mechanical properties, great corrosion resistance, and superb biocompatibility, Ti-based MBs are traditionally used as inert implants for mechanical supports or constraints in orthopedics (joint replacement [11], postoperative fracture/bone defects [12], dentistry [13], and cardiovascular applications [14,15]). However, emerging studies reveal their unique advantages in oncotherapy [16]. Surface-engineered Ti-based MBs can function as bioactive platforms for different physical or chemical antitumor processes [17,18]. To date, the antitumor and anti-recurrence efficacy of modified Ti-based implants has been demonstrated in multiple cancers, including osteosarcoma [19,20], cervical carcinoma [21,22], breast carcinoma [23], hepatocellular carcinoma [24], colorectal cancer [25], alimentary tract tumors [26], gallbladder cancer [27,28], esophageal cancer [29], and ovarian cancer [30].

This review examines the Ti-based MBs developed for oncotherapy applications. By categorizing their antitumor strategies as physical and chemical approaches, the influence of functionalized materials on tumor cell fate and tumor progression was discussed. In addition, we also state the challenges and perspectives of Ti-based MBs for future clinical applications.

## 2. Tumor Hallmarks and the Classification of Antitumor Approaches for Ti-Based MBs

A tumor is a collection of cancer cells and “surrounding” stromal bio-entities [31]. A tumor may be benign or malignant, and a benign one may become malignant in some cases [32]. The eight core hallmarks of malignant tumors include the acquired capabilities for resisting cell death, deregulating cellular metabolism, sustaining proliferative signaling, evading growth suppressors, avoiding immune destruction, enabling replicative immortality, tumor-promoting inflammation, activating invasion and metastasis, inducing or accessing vasculature, and genome instability and mutation [33]. These hallmarks are closely related to the tumor microenvironment, which consists of the aberrant extracellular matrix (ECM), blood vessels, immune cells, carcinoma-associated fibroblasts, and signaling molecules and cytokines [34]. The tumor microenvironment usually shows relatively low pH values, low oxygen contents, high reactive oxygen species (ROS) levels, and immunosuppression [35]. Metabolic reprogramming toward aerobic glycolysis (i.e., the Warburg effect) leads to much higher glucose consumption than normal tissues, accompanied by lactate-driven acidification of the tumor microenvironment [36,37]. Hypoxia of the tumor microenvironment is a consequence of abnormal angiogenic patterning, and it activates the hypoxia-inducible factor-1 (HIF-1) signaling pathway to drive therapeutic resistance and immunosuppressive cytokine secretion [38]. The weakly acidic and hypoxic tumor microenvironment exacerbates mitochondrial oxidative stress, leading to high ROS levels and genomic instability [39]. The immunosuppression of the tumor microenvironment modulates tumor growth and metastasis [40]. In addition, tumors exhibit thermal vulnerability because inefficient vascular networks hinder heat dissipation [41].

Considering the above-mentioned biological characteristics of tumors, various strategies were reported to functionalize Ti-based MBs for antitumor applications, as shown in Table 1. Although these surface modifications on Ti-based MBs inevitably increase manufacturing costs and process complexity, the resulting biofunctionalized implants exhibit superior properties and clinical performance compared to traditional inert Ti implants. Generally, these antitumor functionalizations can be classified into physical modulation strategies and chemical intervention strategies. Physical approaches usually employ external energy fields (e.g., light and electric fields) for localized tumor ablation, due to tumors’ inherent thermal vulnerability [42] and mechanical susceptibility [43], while chemical strategies introduce antitumor chemicals (such as chemotherapeutic drugs and metal ions) onto Ti-based MBs by surface modifications and achieve controlled local chemical delivery by either internal stimuli (i.e., the acidic, hypoxic, and redox-imbalanced tumor microenvironment) or external stimuli (e.g., electromagnetic fields, near-infrared (NIR) light, and ultrasound) [44,45]. It is noteworthy that this dichotomy is only related to antitumor mechanisms rather than surface modification techniques.

## 3. Physical Antitumor Approaches

### 3.1. Photothermal Therapy

The photothermal effect, characterized by light-to-heat conversion via photothermal agents, can be used for antitumor [55,56], antibacterial [57], bone regeneration [58], and other biomedical applications. NIR light is widely used in oncology due to its good penetration depth. Photothermal materials like BP nanosheets, organic polymers, carbon-based materials, and metal nanoparticles can convert NIR light into heat, effectively killing tumor cells through localized hyperthermia. Phototherapy induces endogenous apoptosis in cells through pathways such as increasing the ratio of Bax/Bcl-2 and activating caspase-3. At the same time, heat shock proteins (HSPs) are activated to enhance the effectiveness of other treatment methods due to rising temperatures [59,60].

**Figure 1:**
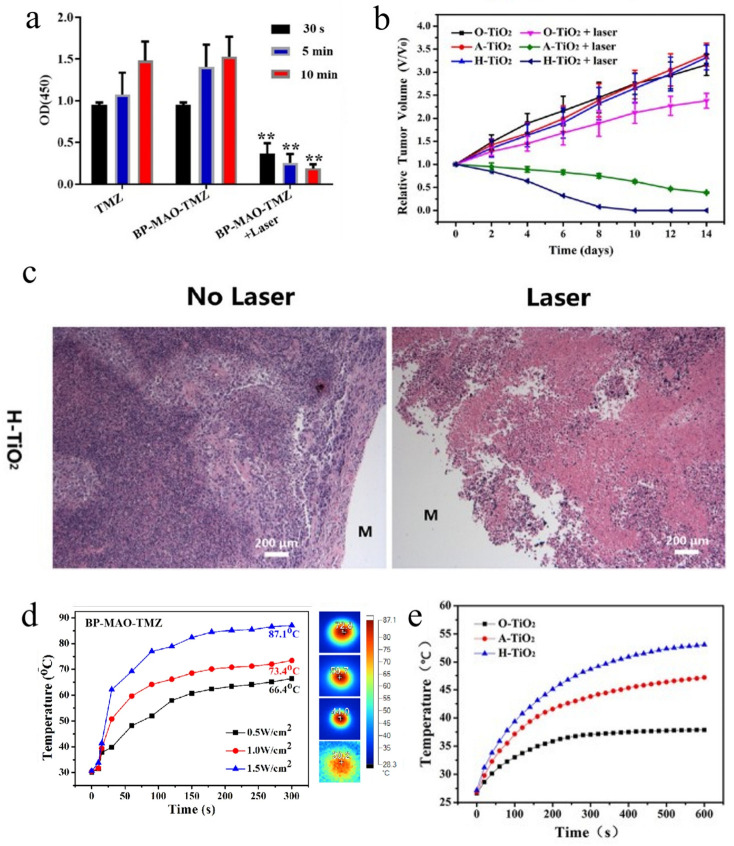
Representative antitumor effect of Ti-based MBs for osteosarcoma. (**a**) CCK-8 results of TMZ and BP-MAO-TMZ with different irradiation durations (** *p* < 0.01) [52]. (**b**) Relative tumor volume variation curve in six groups with increasing days (*n* = 4) [17]. (**c**) Hematoxylin and eosin (H&E) staining images of tumor tissue-implanted material (M represents implanted coating) [17]. (**d**) Photothermal heating curves for different powers of TMZ material [52]. (**e**) Photothermal heating curves of Ti-6Al-4V implant [17] (*n* = 3, mean).

Several attempts have been performed on Ti-based MBs with photothermal coatings for antitumor applications. As shown in Figure 1a, BP-coated TMZ implants exhibit significant tumor inhibition effects via photothermal activation in vitro [52]. Meanwhile, Figure 1b,c present the H-TiO_2_-modified Ti-6Al-4V implants, showing significant efficacy for osteosarcoma therapy in vivo [17]. Although both materials exhibit substantial anti-osteosarcoma efficacy, a critical distinction lies between their photothermal performances. The photothermal heating profiles (Figure 1d,e) demonstrate that BP has a significantly higher photothermal conversion efficiency compared with H-TiO_2_ on Ti-based MBs. This temperature discrepancy highlights an important trade-off: Higher thermal output enhances tumor ablation, but it risks collateral damage to adjacent healthy tissues. Consequently, antitumor Ti-based MBs with preferable photothermal coatings should exhibit a moderate photothermal conversion efficiency with well-controlled NIR irradiation time characteristics to balance the effective tumor suppression (validated in both in vitro and in vivo models) with reduced thermal toxicity.

### 3.2. Photodynamic Therapy

**Figure 2:**
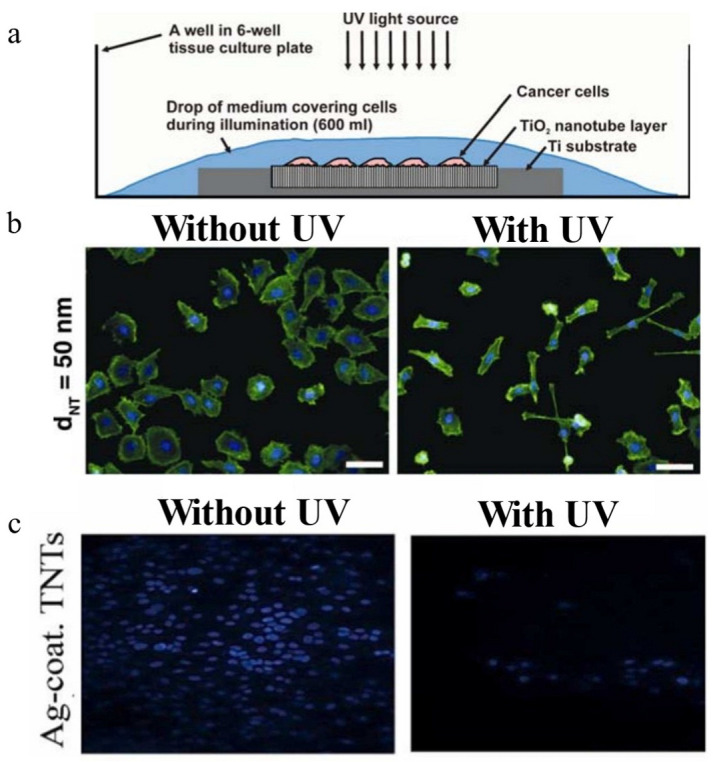
Representative antitumor effect of PDT. (**a**) A schematic of the photocatalytic tumor-killing setup. (**b**) Fluorescence images comparing human cervical carcinoma cell (HeLa cell) morphology on 50 nm diameter TNT with versus without UV irradiation (scale bars: 50 µm) [21]. (**c**) Comparative fluorescence microscopy images of MCF-7 cell populations on Ag-coated TNT with versus without UV irradiation [23].

Photosensitizers can be activated by specific light wavelengths for photodynamic therapy (PDT). The generated cytotoxic ROS, including hydrogen peroxide (H_2_O_2_), hydroxyl radicals (·OH), and superoxide anions (O_2_^−^), induce cancer cell death via oxidative stress [61]. Titanium dioxide (TiO_2_), which is a prominent photocatalytic material, exhibits dual redox activity under ultraviolet (UV) irradiation, producing oxidizing radicals such as ·OH, O_2_^−^, and singlet oxygen (^1^O_2_) [62]. These radicals can trigger tumor cell death through combined apoptotic and necrotic pathways and induce immunogenic cell death (ICD) to activate T cells and antitumor immune response [60]. As shown in Figure 2a,b, Kalbacova et al. [21] fabricated anodized TNT layers on Ti implants. It shows the dose-dependent cytotoxicity against human cervical carcinoma cells under low-intensity UV exposure. Zandvakili et al. [23] further enhanced this strategy by integrating silver (Ag), via deposition, doping, and coating methods, into TNT systems, systematically evaluating their efficacy in suppressing human breast carcinoma cell (MCF-7 cell) proliferation under UV activation (Figure 2c). Quantitative analysis reveals a significant reduction in tumor cell viability post-irradiation.

Compared to pristine TNT, Ag-functionalized TNT exhibits better photodynamic efficacy by generating more ROS under UV irradiation, leading to directly damaging tumor cell membranes and intracellular components. This enhancement should be attributed to Ag’s role, i.e., acting as an electron sink to suppress charge recombination in TiO_2_, amplifying ROS generation. These findings highlight the synergistic potential of metal-TiO_2_ composites in antitumor Ti-based MBs for PDT.

### 3.3. Irreversible Electroporation

IRE, a non-thermal ablation technique, employs high-intensity, ultrashort electric pulses to create irreversible nanopores in cell membranes, triggering caspase-mediated apoptotic pathways, mediating the release of DAMPs, and inducing ICD [63] to achieve precise tumor eradication [64]. He et al. [65] have reported that IRE increases the release of high mobility group box protein B1 (HMGB1). HMGB1 binds to the receptor for advanced glycation end-products, activates the MAPK-p38 pathway, and leads to M1 macrophage polarization. Clinically, IRE has been applied broadly in oncology, such as prostate [66], pancreatic [67], and hepatic [68] cancers. Gold [69], stainless steel [66], and platinum/iridium [68] have been used as electrode materials in IRE because of their good electrical conductivity.

**Figure 3:**
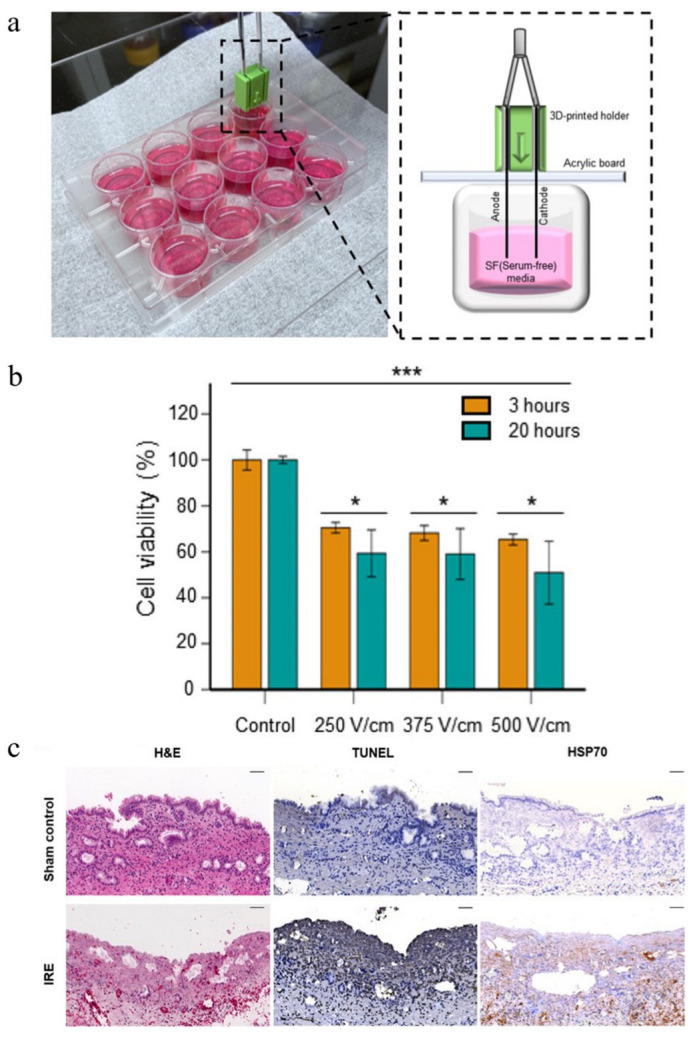
Cytotoxicity and cell viability evaluation of IRE using a chemically polished Ni-Ti alloy wire electrode. (**a**) Schematic of the IRE procedure applied to mouse colon cancer cells (CT-26 cells) with a bipolar wire electrode. (**b**) In vitro cytotoxicity assay results under varying electric field strengths (* *p* < 0.05, *** *p* < 0.001, *n* = 3). (**c**) Histopathological analysis of tumor tissues, including H&E, Terminal dUTP Nick End Labeling (TUNEL), and heat shock protein 70 (HSP70) staining, was conducted at ×20 magnification (scale bar: 50 μm). The results demonstrated IRE-induced cellular damage and apoptosis [26].

Ni-Ti exhibits excellent conductivity, biocompatibility, and shape memory properties, enabling its dual function as implantable stents and IRE electrodes for antitumor applications [25]. Kim et al. [26] fabricated a Ni-Ti stent electrode for gastrointestinal tumor therapy. As illustrated in Figure 3, the chemically polished Ni-Ti wires transmit controlled electric fields to tumor tissues, inducing membrane permeabilization and subsequent apoptosis. In vitro and in vivo studies validated the system’s remarkable tumor suppression efficacy. The cell death rates increased according to the increase in electric field strength. This bifunctional design-simultaneously providing structural reinforcement and localized electroporation, represents a paradigm shift in Ti-based MBs monotherapy, particularly for cancers requiring mechanical support and localized treatment.

Table 2 summarizes the above-mentioned studies on physical antitumor approaches of Ti-based MBs.

## 4. Chemical Antitumor Approaches

The chemical antitumor effects of Ti-based MBs are primarily mediated by drug-loading strategies, whereby therapeutic agents are immobilized onto the material to establish an LDD system [4,70]. These systems induce tumor cell death through elevated local drug concentrations while simultaneously reducing systemic toxicity associated with drug administration [8,20]. LDD systems are broadly categorized into two classes: injection-based and implantable platforms [71]. Injection-based localized drug administration, exemplified by nanoparticle formulations, involves direct tumor-adjacent drug deployment without sustained retention mechanisms [72]. The selective accumulation of nanoparticles in tumor tissues due to vascular leakage and impaired lymphatic drainage, termed the enhanced permeability and retention (EPR) effect, has long been regarded as the cornerstone mechanism for passive tumor targeting [73]. However, accumulating evidence demonstrates substantial heterogeneity in EPR efficacy across tumor subtypes and individual patients, with significant temporal variations even within the same tumor tissue [74]. This intrinsic variability compromises the reproducibility and clinical translatability of nanomaterial-based therapies, particularly in contexts requiring predictable pharmacokinetics [7]. Given these challenges, the direct integration of pre-approved pharmaceutical agents into implants for LDD may represent a more reliable alternative from a clinical safety perspective.

Implantable LDD systems aim to prolong drug retention within tumor-proximal regions [75]. For chemotherapeutic agents that specifically target and eliminate tumor cells during particular cell cycle phases, extended exposure durations are critical [76]. Drug molecules released from implantable systems demonstrate direct exposure to neoplastic cells or the tumor microenvironment, contrasting with injection-based modalities that facilitate systemic drug distribution [71]. This localized delivery mechanism substantially reduces rapid systemic drug leakage, thereby mitigating off-target effects [77]. While polymer-based drug delivery systems have been extensively investigated for implantable LDD applications [78], their relatively limited mechanical performance compared to metallic biomaterials suggests potential future clinical applications through hybrid systems combining both material classes. In orthopedics and maxillofacial surgery, where Ti-based MBs implants are clinically predominant, Ti-based MBs LDD systems demonstrate high translational potential due to their compatibility with established medical device standards. To address the heterogeneous tumor microenvironment, some Ti-based LDD systems are capable of responding to either internal microenvironmental stimuli or external stimuli [79]. These smart release platforms enable spatiotemporal control over therapeutic payloads, including chemotherapeutic agents, immunomodulators, and bioactive metal ions, thereby enhancing therapeutic precision and demonstrating significant clinical translation potential.

### 4.1. Diffusion-Controlled LDD Systems

**Figure 4:**
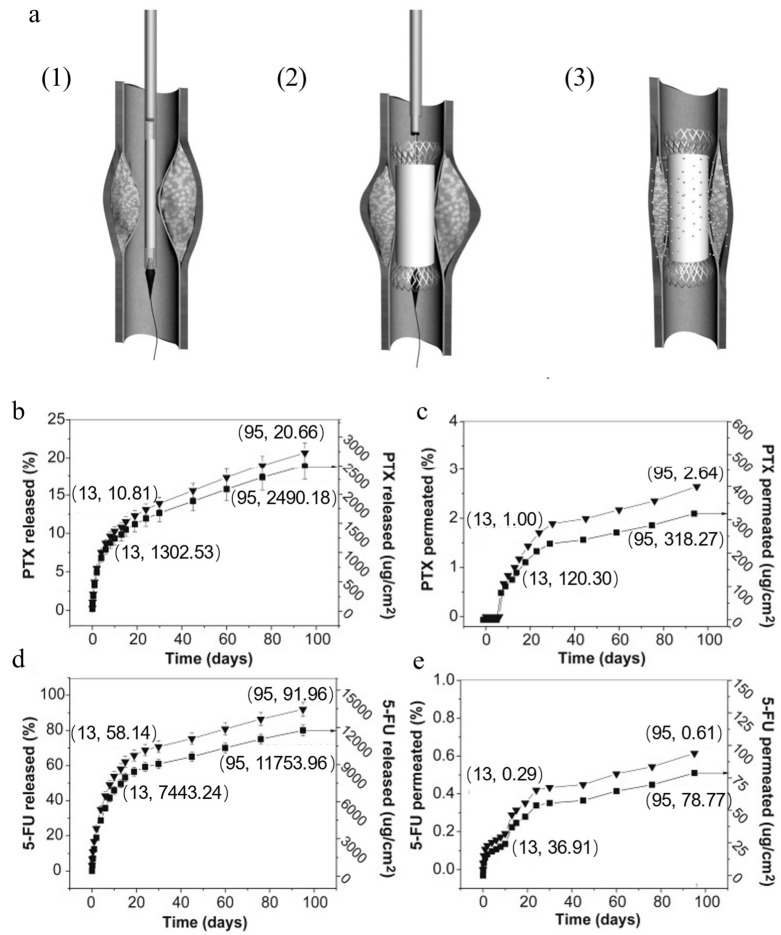
The schematic and drug release profiles of the PTX and 5-FU stent combinations. (**a**) Functional illustration of the antitumor drug/stent combination deployment: (1) implantation into the esophageal tumor site via guidewire, (2) stent expansion, and (3) sustained unidirectional drug release toward tumor tissue. (**b**) Cumulative release profiles of PTX and (**d**) 5-FU from the drug-loaded stent layer. (**c**) Permeation kinetics of PTX and (**e**) 5-FU through the stent backing layer (in vitro drug release in PBS) [82].

**Figure 5:**
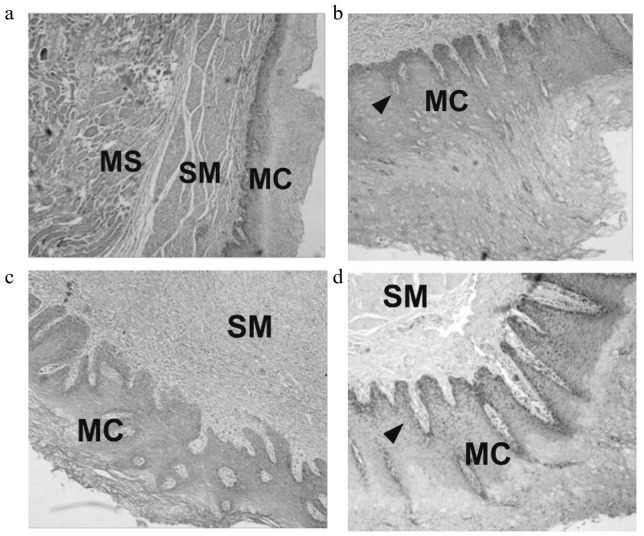
In vivo evaluation of biocompatibility that pathologic images (H&E staining × 10) of porcine esophageal tissue in contact with stents. (**a**) and (**b**) PTX/stent at 13 and 120 days, respectively; (**c**) and (**d**) 5-FU stent at 13 and 120 days, respectively. (Mucosa (MC); submucosa (SM); muscular layer (MS); thickened basal cell layer (triangle) [82].

Some early studies on Ti-based LDD systems achieved therapeutic agent release from implant surfaces primarily through passive diffusion mechanisms. Kaur et al. [80] utilized Ti wire implants functionalized with TNTs in a subcutaneous xenograft mouse model, demonstrating sustained TNF-Related Apoptosis-Inducing Ligand (TRAIL) release over four days that induced apoptosis-mediated tumor suppression. The main mechanism is described that TRAIL binds to the DR4 and DR5 receptors on the surface of tumor cells, triggering receptor trimerization and interacting with the Fas-associated death domain (FADD) to form the death-inducing signaling complex (DISC), activating procaspase-8, which, in turn, activates the mitochondrial pathway, promoting the release of cytochrome C, forming the Apaf-1/caspase-9 apoptosome, activating downstream caspase family proteases, and ultimately leading to apoptosis [81]. Cai et al. [25] performed drug delivery precision in a rabbit orthotopic colorectal cancer model through segmented paclitaxel (PTX)-ethylene-vinyl acetate (PTX-EVA) bilayer film, achieving localized rectal drug enrichment with continuous release exceeding three months. Figure 4a depicts the bilayered Ti-based drug-eluting stent model engineered for simultaneous esophageal lumen dilation and localized antitumor therapy. Figure 4b,d present the sustained release kinetics of PTX and 5-fluorouracil (5-FU) from the drug-loaded polymeric layer, respectively. Figure 4c,e demonstrate critically restricted retrograde drug permeation through the permeation-resistant backing layer, with cumulative leakage of both therapeutics remaining below 4%. These data collectively validate the stent’s capacity to spatially confine drug diffusion to the tumor microenvironment, thereby minimizing systemic dispersions. Similarly, Liu et al. [82] validated a dual-drug polymer-coated esophageal stent (PTX/5-FU stent) in a porcine large-animal model. As shown in Figure 5, the in vivo biocompatibility of PTX and 5-FU stents following implantation in porcine esophageal tissue was also assessed. The H&E staining results reveal no significant granulation tissue formation or inflammatory response in the esophageal mucosa adjacent to either the PTX- or 5-FU-coated stent segments.

Although these systems demonstrate progressive improvements in release duration, their drug-release kinetics remain constrained by passive diffusion mechanisms, which are related to drug–carrier physicochemical properties and tumor microenvironmental variables. Critical limitations include an initial uncontrolled burst release, evidenced by rapid TRAIL elution in Kaur’s TNT system, which risks systemic toxicity, alongside static release profiles that lack responsiveness to dynamic tumor microenvironmental shifts such as pH gradients in hypoxic cores of tumors [83]. Furthermore, limited drug penetration persists in dense stromal regions due to elevated interstitial fluid pressure and ECM [84].

Emerging hybrid systems aim to transcend the above-mentioned constraints by internal stimuli-responsive mechanisms or external stimuli ones for spatiotemporal control of chemical release. Beyond endogenous triggers such as pH-sensitive or redox-sensitive polymers, exogenous-actuated platforms leverage NIR light, alternating magnetic fields, or focused ultrasound to enable on-demand drug release. These approaches synergize the anatomical precision of Ti-based MBs with dynamic release modulation, potentially overcoming passive diffusion limitations while maintaining the mechanical advantages of metallic implants for clinical translation.

### 4.2. Internal Stimuli-Responsive LDD Systems

**Figure 6:**
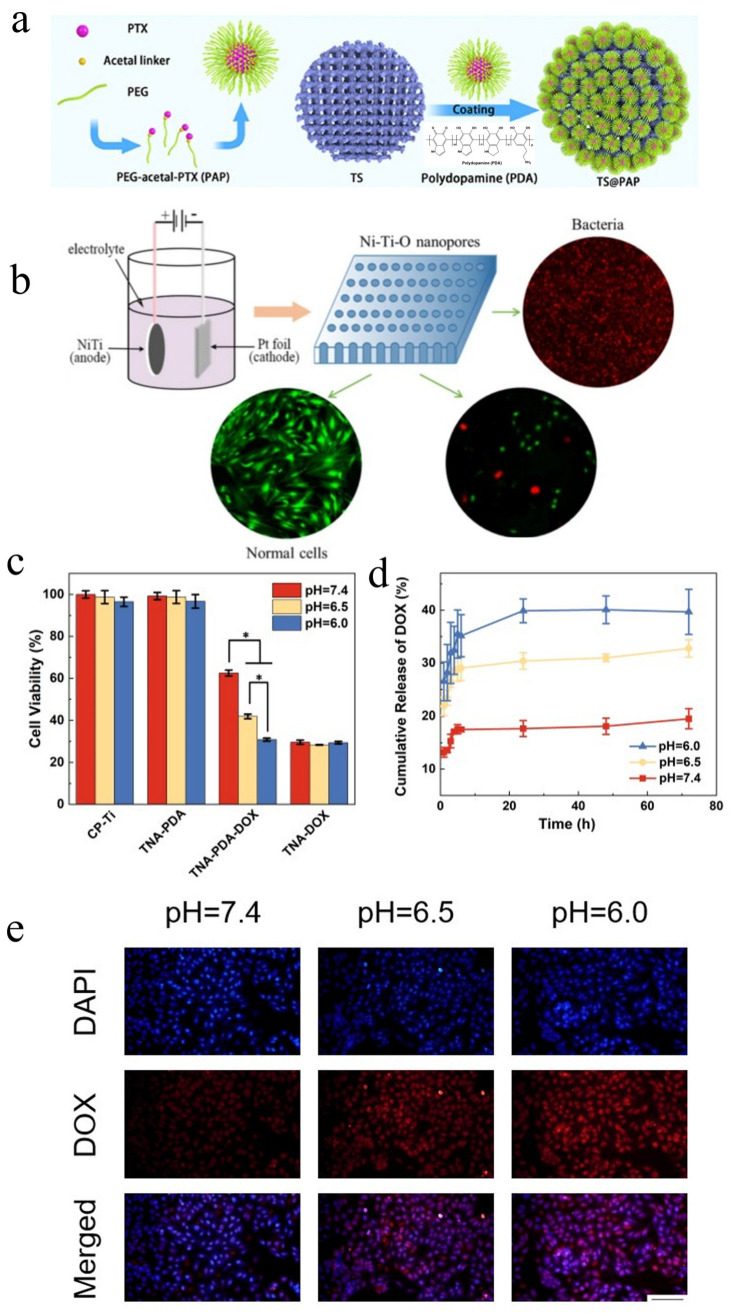
Representative antitumor effects of internal stimuli-responsive LDD Systems. (**a**) Schematic illustration of the composite scaffold of the self-assembly of amphiphilic PEG-acetal-PTX prodrug on the 3D printed TS implants [19]. (**b**) Graphic summary of selective inhibition of Ni-Ti-O nanoporous layer on cancer cells [88]. (**c**) MG63 cell viability after 24 h of indirect culture with samples (* *p* < 0.05). (**d**) pH-dependent drug release profiles (pH = 7.4, 6.5, 6.0; in vitro drug release in PBS). (**e**) Confocal microscopy images of DAPI- and DOX-stained MG63 cells; scale bar: 50 µm [20].

**Figure 7:**
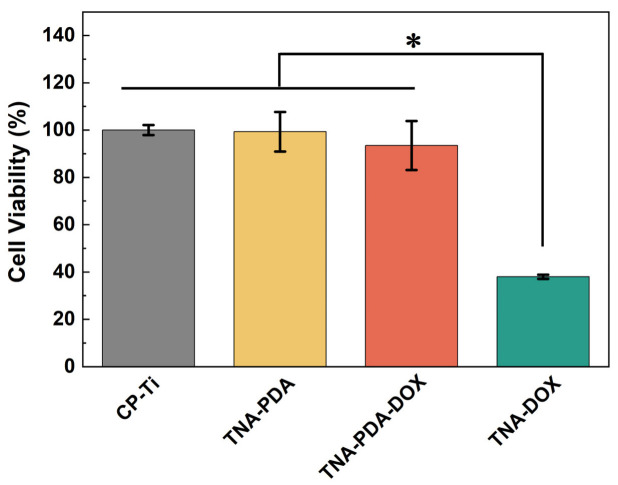
In vitro evaluation of biocompatibility. MTT assay results of the mouse embryo osteoblast precursor cells’ (MC3T3 cells) viability cultured for 24 h on different samples (* *p* < 0.05) [20].

Internal stimuli-responsive LDD systems for tumor therapy are categorized into pH-responsive, ROS-responsive, and enzyme-responsive implants, each exploiting distinct tumor microenvironment features [85]. The pH-responsive systems primarily utilize tumor-associated glycolytic acidosis to achieve spatiotemporally controlled drug release [86]. Tumor cells demonstrate augmented aerobic glycolysis due to excessive nutrient uptake. Glucose undergoes glycolysis and is converted into lactic acid. To maintain intracellular pH homeostasis, tumor cells extrude surplus protons extracellularly. This leads to a decrease in the extracellular microenvironment pH and the formation of an acidic tumor microenvironment. Under normal physiological pH, drugs stay stable, but their release mechanism activates in the acidic tumor microenvironment, enabling precise tumor-site drug delivery. This approach both boosts therapeutic efficacy and reduces side effects on normal tissues [87]. For example, Fan et al. [19] fabricated 3D-printed Ti scaffolds functionalized with polyethylene glycol (PEG)-acetal-PTX prodrug nanoparticles (Figure 6a), where acid-labile linkers enabled 85% PTX release at pH 6.5 over 72 h while maintaining over 93% bone marrow stem cell viability at physiological pH, as validated in subcutaneous osteosarcoma mouse models. Similarly, Liu et al. [88] engineered anodized Ni-Ti-O nanoporous layers through a controlled electrochemical oxidation process (Figure 6b), which demonstrated a pH-responsive selective release of Ni^2+^ ions under acidic conditions (pH 6.0–6.8). This ion-specific elution mechanism induced ROS-mediated apoptosis in cancer cells and exhibited over 90% antibacterial efficacy against Staphylococcus aureus in rat xenograft models. In another approach, Xing et al. [28] engineered calcined layered double hydroxide coatings on Ni-Ti stents to enhance arsenite loading in acidic microenvironments, achieving targeted gallbladder tumor suppression in rabbit femoral defect models with less than 10% drug leakage at neutral pH. Additionally, Xiao et al. [20] introduced PDA-coated TNT arrays on pure Ti (TNA-PDA-DOX) that leveraged the inherent pH-responsive behavior of PDA. Figure 6c reveals the pH-dependent cytotoxicity of TNA-PDA-DOX. Figure 6d illustrates accelerated DOX elution under acidic conditions (>80% cumulative release at pH 6.0 vs. <30% at pH 7.4 after 72 h). Figure 6e visualizes enhanced co-localization of cell nuclei and DOX in acidic microenvironments (pH 6.0), evidenced by merged 4’,6-diamidino-2-phenylindole (DAPI)/DOX signals, while physiological pH (7.4) shows minimal nuclear penetration. Furthermore, the in vitro biocompatibility of samples was also assessed. As shown in Figure 7, the cell viability of TNA-PDA (~99%) and TNA-PDA-DOX (~93%) is not significantly different from that of CP-Ti (the control group).

The ROS-responsive systems exploit elevated H_2_O_2_ concentrations in tumor microenvironments to drive therapeutic actions [85]. A representative example is the work by Yan et al. [89]. A zirconium-based metal–organic framework film, responsive to endogenous H_2_O_2_ levels, was introduced on Ti implants. This system utilized H_2_O_2_-triggered Fenton reactions to convert Fe^2+^ to Fe^3+^, modulating surface charge to accelerate DOX release via electrostatic interactions. When combined with auxiliary NIR irradiation to enhance therapeutic precision, the endogenous ROS-responsive system achieved 92% tumor suppression in subcutaneous osteosarcoma-bearing mouse models. Post-treatment analysis revealed Fe^3+^-mediated superhydrophilic conversion with contact angles below 10 degrees, enhancing protein adsorption and osteogenic differentiation. The sustained release of Fe ions over seven days synergistically upregulated osteogenic markers (OCN, RUNX2) in bone marrow stromal cells, as evidenced by 2.1- to 3.3-fold increases in ALP and Runx2 expression compared to untreated Ti controls.

Despite the absence of reported enzyme-responsive antitumor LDD systems on Ti-based MBs, such platforms hold significant therapeutic potential due to the pathological enzymatic activity inherent to tumor microenvironments. Wang et al. [90] developed a dual-enzyme (ALP/MMP-2)-responsive peptide self-assembly system for spatiotemporally controlled co-delivery of chemotherapeutic HCPT and immunomodulatory IND. The system selectively disassembles in tumor microenvironments with dual-enzyme overexpression, releasing IND to activate antitumor immunity while forming cytotoxic nanofibers for targeted chemotherapy. Wu et al. [91] engineered a trypsin-responsive bioactive peptide (1-Pept) that co-assembled with DOX into enzyme-instructed nanofibers (1-Pept/Dox NFs). Intracellular trypsin triggered sequential transformations: Initial NF disassembly released DOX for nuclear DNA damage, while residual peptide fragments reassembled into dense secondary NFs, disrupting cytoskeletal integrity and activating caspase-3-mediated apoptosis. These systems for tumor therapy exhibit superior clinical translation potential compared to diffusion-controlled LDD approaches through their ability to exploit intrinsic pathological features of the tumor microenvironment for precise therapeutic control. This suggests that developing enzyme-responsive tumor therapeutic systems on Ti-based MBs holds significant research potential.

### 4.3. External Stimuli-Responsive LDD Systems

The inherent limitations of conventional nanotherapeutic approaches encompassing traditional chemotherapeutics, molecularly targeted agents, immune-based therapies, and physical modalities stem from tumor heterogeneity and microenvironmental constraints. Chemotherapeutic drugs exhibit suboptimal penetration in dense stromal regions, exacerbated by elevated interstitial fluid pressure and extracellular matrix barriers, whereas physical ablation techniques often fail to eradicate residual tumor tissues, increasing recurrence risks [92,93]. Targeted therapies, though effective in suppressing specific oncogenic pathways, frequently induce compensatory signaling cascades such as the c-Mesenchymal-Epithelial Transition factor (MET) or AXL Receptor Tyrosine Kinase (AXL) upregulation, enabling tumor escape mechanisms [94,95,96]. Immune-based strategies are hindered by immunosuppressive niche features, including regulatory T-cell infiltration and PD-L1 overexpression [97]. Collectively, these challenges underscore the inadequacy of single-modality interventions in addressing the spatiotemporal adaptability of malignancies.

To overcome these limitations, emerging therapeutic paradigms integrate exogenous stimuli-responsive drug delivery systems with synergistic mechanisms. Such strategies mitigate resistance by simultaneously disrupting primary oncogenic drivers [98,99,100] and compensatory survival pathways [101,102], enhancing therapeutic indices through tumor-selective activation and dose de-escalation, and enabling precision targeting of evolving malignant subclones. Recent advancements in Ti-based MBs exemplify the translational potential of this approach. Engineered to release therapeutic agents in response to exogenous stimuli, these implants combine localized cytotoxicity with microenvironmental modulation, achieving spatially controlled tumor suppression while minimizing off-target effects. By harmonizing stimulus-triggered drug release with physical or biochemical tumor disruption, these platforms demonstrate superior efficacy in eradicating residual disease compared to conventional monotherapies, thereby addressing both microenvironmental barriers and tumor adaptability through a unified mechanistic framework.

**Figure 8:**
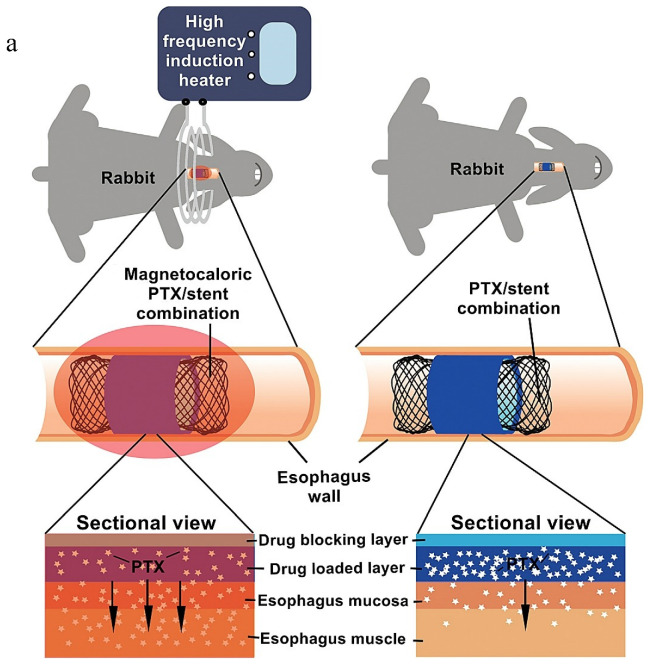

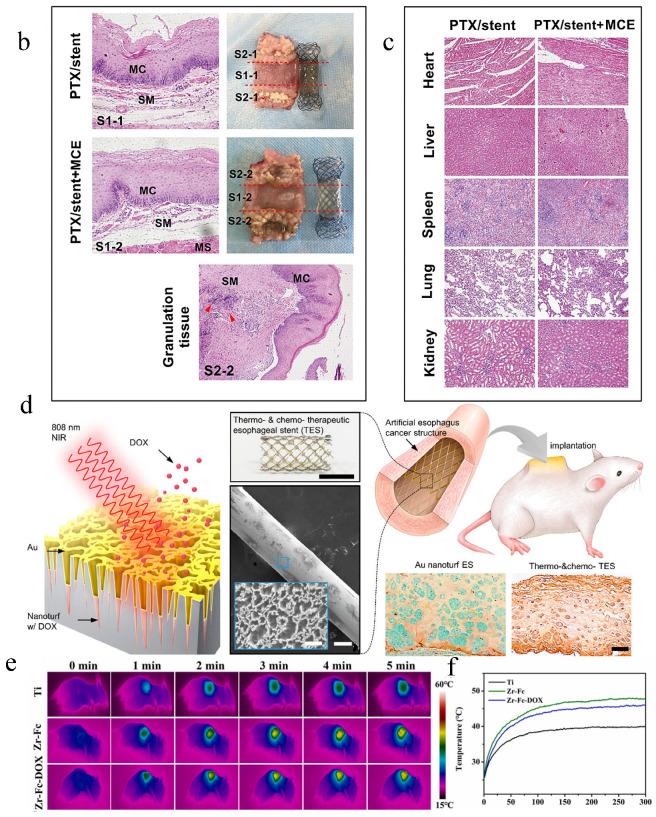
Representative antitumor effects of external stimuli-responsive LDD Systems. (**a**) Schematic illustration of A PTX/Ni-Ti stent combination with temperature-responsive phase-change 1-hexadecanol for magnetocaloric drug delivery [25]. (**b**) Pathological and anatomical images of esophageal tissues (S1: stent film-covered segment, S2: bare stent segment) with and without MCE. H&E magnification: 200 for S1-1 and S1-2, 100 for S2-2. MC: mucosa, SM: submucosa, MS: muscular layer, red arrow: inflammatory infiltration [25]. (**c**) Systemic safety evaluation via H&E-stained images (×200) of major organs 15 days post-stent implantation [25]. (**d**) Design of the thermo-chemotherapeutic esophageal stent (TES) using DOX/Au-coated nanoturf structures [103]. (**e**) Thermal imaging of surface temperature variations across samples during NIR irradiation [89]. (**f**) The temperature curve of different samples under NIR irradiation [89].

External stimulus-responsive LDD Ti-based MB systems aim to achieve precise spatiotemporal control of therapeutic release through external triggers such as electromagnetic fields, NIR light, or ultrasound, thereby enhancing treatment specificity and minimizing systemic toxicity [4]. Although current research on such Ti-based systems remains limited, their non-invasive triggering mechanisms and programmable drug release profiles hold significant potential for clinical applications, particularly in preventing tumor recurrence. For instance, Jin et al. [29] developed a Ni-Ti stent coated with a temperature-sensitive bilayer film containing PTX and phase-change 1-hexadecanol. Figure 8 shows the antitumor efficacy and biocompatibility of a magnetocaloric effect (MCE)-activated, PTX-loaded Ni-Ti stent. Figure 8a illustrates a temperature-responsive drug delivery system combining PTX with a phase-change material (1-hexadecanol) on a Ni-Ti stent. Under alternating magnetic fields, the MCE generates heat to melt 1-hexadecanol, enabling on-demand PTX release while maintaining structural stability via a drug-blocking layer. Figure 8b reveals distinct tissue responses in esophageal segments: the PTX/stent+MCE group (S1-1/S1-2) exhibits reduced inflammatory infiltration (red arrows) in the MC and SM under 200× magnification, contrasting with granulation tissue formation and MS disruption in the bare stent region (S2-2) of non-activated groups. Figure 8c shows no pathological abnormalities in either PTX/stent or PTX/stent+MCE groups (200×), confirming the localized therapeutic action and minimal off-target toxicity. Similarly, Lee et al. [103] engineered a multifunctional gold nanoturf-coated esophageal stent (Au nanoturf ES) for synergistic photothermal/chemotherapeutic applications in artificial esophageal cancer models implanted in BALB/c nude mice (Figure 8d). Au nanoturf ES exhibits excellent photothermal properties, enabling explosive drug release upon near-infrared irradiation. Furthermore, its structure features anti-cell adhesion. This reduces the cell contact area, diminishes the function of adhesion-related proteins like αβ integrin, inhibits the formation of long filopodia in tumor cells, and disrupts cell spreading and seeding, thereby demonstrating a significant antitumor effect. Comparative histology reveals extensive tumor apoptosis (brown TUNEL+ staining) in the DOX-loaded thermo-chemotherapeutic esophageal stent (TES) group under NIR irradiation (0.4 W cm^−2^), contrasting with viable tumor clusters (blue nuclei) in non-therapeutic controls, highlighting the platform’s synergistic efficacy in localized cancer treatment. Yan et al. [89] further constructed this paradigm with a dual-stimulus-responsive Zr-MOF film on Ti substrates. The Zr-MOF membrane has excellent photothermal properties. Fe^2+^ in Zr-MOF membrane can undergo a Fenton reaction with H_2_O_2_ in the tumor microenvironment and be converted into Fe^3+^, reducing the negative charge of the Zr-MOF membrane, releasing DOX, and making the Zr-MOF membrane change from hydrophobic to hydrophilic. Studies indicate that the hydrophilic titanium surface can remarkably upregulate the gene expression levels of integrin subunits β1 and α_v_, enhancing cell adhesion and thereby promoting osteogenic differentiation [104]. Figure 8e,f evaluated the photothermal conversion efficiency of Ti, Zr-Fc, and Zr-Fc-DOX under 808 nm NIR irradiation using infrared thermal imaging and temperature–time profiles, demonstrating the superior photothermal responsiveness of Zr-Fc-DOX. In a subcutaneous 143B osteosarcoma mouse model, NIR irradiation synergized with endogenous H_2_O_2_ to enhance DOX release via photothermal disruption of hydrophobic interactions and redox-driven modulation of surface hydrophilicity. This dual-triggered system not only suppressed tumor growth but also promoted post-treatment osteogenic gene expression, highlighting its capacity for sequential therapy/regeneration functionality. Critically, these systems are designed to remain pharmacologically inert until activated by external stimuli, ensuring “on-demand” drug release during tumor recurrence while maintaining long-term silent states otherwise. While electromagnetic and NIR triggers dominate current studies, ultrasound, with its non-invasiveness and deep tissue penetration, represents an underexplored yet promising external stimulus for future Ti-based LDD implants. Collectively, these innovations underscore the potential of exogenous stimulus-responsive Ti alloys to bridge precision oncology and functional restoration, offering dynamic, context-aware therapeutic interventions tailored to tumor recurrence patterns.

Table 3 summarizes the above-mentioned studies on chemical antitumor approaches of Ti-based MBs.

## 5. Challenges and Perspectives

Traditional tumor treatments, i.e., chemotherapy, radiotherapy, and surgical resection, still show certain limits, including high surgical risk and tissue defects during treatment, frequent drug administration and high total dose, and incomplete obliteration of tumor cells. Consequently, highly selective and localized treatments with lower side effects are urgently needed. Antitumor Ti-based MBs demonstrate their great potential for further oncology clinical applications. However, both physical and chemical approaches exhibit inherent limitations, as shown in Table 4. Physical methods offer advantages in controllable therapeutic intensity/duration and generally favorable long-term biocompatibility of materials, rendering them relatively safer during treatment. Their critical drawback lies in the insufficient spatial precision of exogenous therapeutic approaches utilizing antitumor Ti-based MBs when targeting intact tumor masses. Confronted with complex heterogeneous tumor microenvironments, this spatial imprecision may result in incomplete tumor eradication during treatment, subsequently inducing iatrogenic dissemination of residual malignant cells. In contrast, chemical methods demonstrate superior comprehensive tumor-killing efficacy and tumor-targeting capacity. However, their fundamental limitation resides in the difficulty of achieving complete control over therapeutic agent release. Even if endogenous response mechanisms can initiate drug release, the subsequent “uninterrupted” drug elution post-implantation, rather than externally switchable release modalities, may provoke systemic toxicity risks. In addition, biosafety is always the most important consideration for an implant material. Since the surface modifications on Ti-based MBs inevitably introduce various chemicals for either external stimulus-responsive therapy or building the LDD systems, most components (except therapeutic agents) should be biocompatible. In the future, more attention should be paid to the development of more intelligent antitumor Ti-based MBs with more controllable and localized release of external energy fields or therapeutic chemicals. For instance, the enzyme-responsive antitumor LDD Ti-MBs may probably show great potential. In addition, the antitumor Ti-MBs scaffolds showing immunomodulated anti-tumor activity also deserve consideration [105]. Titanium oxide nanoparticles have been reported to have the ability to initiate the tumor immune cycle through the induction of ICD [106,107]. Spherical metal–organic frameworks (MOFs) efficiently induce the repolarization of tumor-associated macrophages (TAMs) from the immunosuppressive M2 phenotype to the antitumor M1 phenotype, while simultaneously enhancing dendritic cell (DC) maturation, thereby potentiating antitumor immunity [108,109].

In conclusion, by bridging material science with oncology, this work summarizes the transformative potential of Ti-based MBs in developing next-generation, multifunctional antitumor platforms, offering insights for both academic and clinical communities.

## Figures and Tables

**Table 1:** Properties and applications of Ti-based MBs.

The Substrates of Ti-Based MBs	Surface Modification	Properties	Application	Ref.
Commercial pure Ti (CP-Ti)	Electrochemical anodization	Excellent biocompatibility, high orientation, and large surface area with tunable pore sizes	Self-organized TiO_2_ nanotube (TNT) can be used for photo-induced cancer cell killing	[21,46,47]
	Polydopamine (PDA)-coated TNT	Good biocompatibility, strong adhesive properties, and sensitive pH-responsiveness	Polydopamine-coated TNT arrays on pure Ti (TNA-PDA-DOX) have a good anti-osteosarcoma function by the pH-responsive release of Doxorubicin (DOX)	[20,48]
	Ti substrate with ZnO nanoparticles decorated with naringin	Surface nano-treatment enhances the response of osteoblasts	Ti substrate with pH-responsive naringin ZnO nanoparticle promotes the construction of osteosarcoma resection	[49]
Ti-6Al-4V	Plasma-sprayed CaO-MgO-SiO_2_	Excellent biocompatibility, corrosion resistance, and mechanical properties	Plasma-sprayed CaO-MgO-SiO_2_ bioactive glass–ceramic coatings on Ti-6Al-4V alloy for bone regeneration	[17,50]
	Hydrogenated black titanium dioxide (H-TiO_2_) modified 3D printed Ti-6Al-4V	Porous features favor bone ingrowth.	H-TiO_2_-modified Ti-6Al-4V implants for photothermal therapy of bone tumor and bone regeneration	[17,51]
Ti-12Mo-10Zr (TMZ)	Black phosphorus (BP)-coated TMZ	Low elastic modulus, appropriate compressive yield strength, and high plasticity	BP microarc oxidation (MAO) TMZ implant inhibits osteosarcoma cancer cells under the irradiation of NIR	[52]
Nickel-titanium (Ni-Ti)	Chemically polished Ni-Ti wire	Excellent conductivity, biocompatibility, and shape memory properties	Irreversible electroporation (IRE) using chemically polished Ni-Ti wires induces cancer cell death	[26,29]
TiO_2_ nanoparticles	TiO_2_ nanoparticles doped with fluorine (F)/PDA/collagen	Excellent near-infrared-activated photothermal and photocatalytic properties	TiO_2_@F/PDA/collagen nanoparticles anchored on Ti surfaces eliminate osteosarcoma cells and promote osteogenic differentiation of BMSCs	[53,54]

**Table 2:** Physical modulation strategies of Ti-based MBs for antitumor applications.

Therapeutic Strategy	Ti-Based MBs	In Vitro Studies	In Vivo Studies	Ref.
Photothermal therapy	BP-coated TMZ implant; H-TiO_2_ coating on Ti-6Al-4V implant	CCK8 assay test cytocompatibility and bone regeneration; photothermal effects for bone tumor cells	H&E staining; relative tumor volume variation curve	[17,52]
Photodynamic therapy	Self-organized TNT; Ag-deposited, doped, and coated titanium dioxide nanotubes	Photocatalytic experiment; cytocompatibility and proliferation	-	[21,23]
Irreversible electroporation	Chemically polished Ni-Ti alloy wire	CCK8 assay test cytotoxicity and viability of CT-26 cancer cells	H&E, TUNEL, and HSP70 staining of tumor tissues	[26]

**Table 3:** Chemical modulation strategies of Ti-based MBs for antitumor applications.

Therapeutic Strategy	Ti-Based MBs	In Vitro Studies	In Vivo Studies	Ref.
Diffusion-controlled LDD system	PTX or 5FU/nitinol stent	Drug release and permeation	H&E staining; pig body weight changes	[25]
Internal stimuli-responsive LDD system	3D-printed TS- and pH-responsive PEGylated paclitaxel prodrugs; Ni-Ti-O nanoporous layers; double hydroxides (LDHs) layer on nitinol; TNA-PDA-DOX	Live/dead staining assay; cell viability assay; pH-dependent drug release profiles	-	[19,20,88]
External stimuli-responsive LDD systems	A PTX/nitinol stent combination with temperature-responsive phase-change 1-hexadecanol; DOX/Au-coated nanoturf structures; TiO_2_ nanoparticles doped with F/PDA)/collagen; a dual-stimuli-responsive Zr-MOF film on Ti substrates.	Osteogenic differentiation of BMSCs; antitumor ability assay; photothermal effect	H&E staining; thermal images of nude mice under NIR; pathological and anatomical images of esophageal tissues	[29,53,89,103]

**Table 4:** Comparison of physical and chemical approaches across evaluation metrics.

Evaluation Metric	Physical Approaches	Chemical Approaches
Long-term toxicity	Low risk	Potential risk
Therapeutic control	Real-time modulation	Release kinetics depend on coating design
Drug resistance	Irrelevant	Possible
Immunization	Controllable	Possible immune suppression

## Data Availability

No new data were created or analyzed in this study.

## Abbreviations

The following abbreviations are used in this manuscript:

Ag	Silver
AXL	AXL Receptor Tyrosine Kinase
BP	Black Phosphorus
CP-Ti	Commercial Pure Ti
CT-26 cells	Mouse Colon Cancer cells
DAPI	4’,6-Diamidino-2-Phenylindole
DC	Dendritic Cell
DISC	Death-Inducing Signaling Complex
DOX	Doxorubicin
ECM	Extracellular Matrix
EPR	Enhanced Permeability and Retention
F	Fluorine
FADD	Fas-associated Death Domain
HeLa cell	Human Cervical Carcinoma cell
HIF-1	Hypoxia-Inducible Factor-1
HMGB1	High Mobility Group Box Protein B1
H_2_O_2_	Hydrogen Peroxide
HSPs	Heat Shock Proteins
HSP70	Heat Shock Protein 70
H-TiO_2_	Hydrogenated Black Titanium Dioxide
H&E	Hematoxylin and Eosin
LDHs	Double Hydroxides
ICD	Immunogenic Cell Death
IRE	Irreversible Electroporation
LDD	Local Drug Delivery
MAO	Microarc Oxidation
MBs	Metallic Biomaterials
MC	Mucosa
MCE	Magnetocaloric Effect
MCF-7 cell	Human Breast Carcinoma Cell
MC3T3 cells	Mouse Embryo Osteoblast Precursor Cells
MET	c-Mesenchymal–Epithelial Transition Factor
MOFs	Metal–Organic Frameworks
MS	Muscular Layer
NIR	Near-infrared
Ni-Ti	Nickel–Titanium
NMPA	National Medical Products Administration
O_2_^−^	Superoxide Anion
^1^O_2_	Singlet Oxygen
·OH	Hydroxyl Radical
OCN, RUNX2	Osteogenic Marker
PDA	Polydopamine
PDT	Photodynamic Therapy
PEG	Polyethylene Glycol
PTX	Paclitaxel
PTX-EVA	Paclitaxel-Ethylene-Vinyl Acetate
PTX/5-FU stent	Polymer-Coated Esophageal Stent
ROS	Reactive Oxygen Species
Saos-2	Osteosarcoma Cell
SM	Submucosa
TAMs	Tumor-Associated Macrophages
TES	Thermo-chemotherapeutic Esophageal Stent
Ti	Titanium
TNA-PDA-DOX	Polydopamine-Coated TNT Arrays On Pure Ti
TNT	TiO_2_ Nanotube
TMZ	Ti-12Mo-10Zr
TRAIL	TNF-Related Apoptosis-Inducing Ligand
TUNEL	Terminal dUTP Nick End Labeling
UV	Under Ultraviolet
5-FU	5-Fluorouracil
